# Contact time and disinfectant formulation significantly impact the efficacies of disinfectant towelettes against *Candida auris* on hard, non-porous surfaces

**DOI:** 10.1038/s41598-023-32876-y

**Published:** 2023-04-10

**Authors:** Maxwell G. Voorn, Alyssa M. Kelley, Gurpreet K. Chaggar, Xiaobao Li, Peter J. Teska, Haley F. Oliver

**Affiliations:** 1 Department of Food Science, Purdue University, West Lafayette, IN 47906 USA; 2 Diversey Inc., Charlotte, NC 28273 USA

**Keywords:** Microbiology, Health care

## Abstract

There has been an increase in *Candida auris* healthcare-associated infections, which result from cross-contamination from surfaces and equipment. In this study, we tested the efficacies of EPA-registered disinfectant towelettes products that are increasingly used for infection control against *C. auris* at a range of contact times following modifications to standard EPA protocol MB-33-00. Hydrogen peroxide (HP)-based disinfectant towelettes were more efficacious against *C. auris* than the quaternary ammonium chloride (QAC)-alcohol-based disinfectant towelettes irrespective of tested contact times. Thirty s contact time was significantly less effective in reducing *C. auris* compared to 1-, 2-, 3-, and 10-min contact times. However, there were no statistically significant differences in the level of disinfection among 1-min and longer contact times regardless of product chemistry. None of the products achieved a standard six-log_10_ reduction at any tested contact times. Overall**,** the HP-based disinfectant towelette was significantly more fungicidal than the QAC-alcohol-based disinfectant towelette. For all product types, 30 s contact time did not achieve the same level of disinfection as 1-min or longer contact times. Overall, disinfectant towelette efficacy is dependent upon product formulation and contact time.

## Introduction

*Candida auris* is a pathogenic yeast^1^ that causes invasive infections resulting in 40% in-hospital mortality^2^. Immunocompromised populations (e.g., premature infants, pregnant women, cancer patients, the elderly)^3^ are at greatest risk of infection and death. *Candida* *auris* persists in healthcare environments for long periods of time in a range of conditions^4^ complicating complete disinfection and extending outbreaks^5^. This specifically threatens intensive care unit (ICU) patients that often have longer stays in healthcare institutions. Recent investigations suggest that one-third to half of all patients on a given unit, especially in a long-term acute care hospital, can become colonized with this fungal pathogen within weeks of an index patient entering the facility^2^. Outbreaks associated with *C. auris* have been difficult to control and in severe cases, can require the temporary closure of hospital units^6^.

Hospital environmental surfaces are known reservoirs of nosocomial pathogens^7^ and contribute at least 20% of HAIs^8^. Surface-level cleanliness and disinfection has shown to be pivotal in controlling the transmission of pathogens within the healthcare setting^7^. Recently, pre-wetted disinfectant towelettes have become a predominant method of disinfecting equipment and environmental surfaces due to their faster disinfection process, higher compliance with disinfection standards, and overall cost savings as compared to traditional disinfection methods^9^. Previous studies by our group suggested that the choice of disinfectant, concentration, and dimensions of surface area wiped can significantly impact the overall disinfectant towelette efficacy against bacteria^10^. Additionally, reduced contact times as compared to label directions can also significantly lower the disinfectant’s efficacy^11^. According to Center for Disease and Prevention (CDC) guidelines on disinfection and sterilization in healthcare facilities^12^, vegetative bacteria and yeast (e.g., *Candida*) can be inactivated by a low-level disinfectant at exposure times of 30–60 s. Most of these disinfectants are registered by Environmental Protection Agency (EPA); the EPA methods are standardized by the Association of Official Agricultural Chemists (AOAC) International^13^. Briefly, this use-dilution method is performed by soaking stainless steel carriers in bacteria followed by disinfectant treatment and enumeration of surviving bacteria in a broth. This standard method is, however, confined to testing two pathogens i.e., *Pseudomonas aeruginosa* and *Staphylococcus aureus*^14^. Several studies highlight the notable limitations of these AOAC use-dilution tests for liquid disinfectants^12^. Although the latest standard EPA MB-35-03 method for testing disinfectant efficacy is extended to include fungal pathogen i.e., *C. auris*^15^, this method is primarily designed for testing liquid antimicrobial test substances including sprays and water-soluble powders for use on hard, non-porous surfaces. There is limited guidance on evaluating towelettes efficacy compared to liquid expressed from towelettes as a means of evaluation, disregarding other factors such as wipe material that could also impact overall performance^16^.

The standard EPA method for disinfectant towelette efficacy testing against bacterial pathogens i.e., *S. aureus*, *P. aeruginosa*, and *Salmonella enterica* utilizes significantly smaller surface sizes (e.g., 25 mm × 75 mm glass slide carriers)^13^, which is not practically applicable to larger surface areas where factors such as evaporation can significantly affect efficacy. Similarly, protocol EPA MB-35-03 is limited to testing disinfectant efficacy using smaller size carriers (one cm in diameter) inoculated with *C. auris*^15^. In our recent study^10^, we demonstrated that surface area wiped, product type, and bacterial strain significantly impact the bactericidal efficacies of ready-to-use (RTU) disinfectant towelettes against *P. aeruginosa* and *S. aureus*. This study gave further insights into the decreased bactericidal efficacies of disinfectant towelettes when larger, more practical surface areas are wiped due to decreasing liquid released per ft^2^ from the towelette. This is an important aspect to consider given EPA does not require disinfectant manufacturers to include a recommended maximum surface area per towelette on their product labels.

Considering the contagiousness and virulence of *C. auris*, it is important to evaluate the efficacy of disinfectant towelettes with varying chemistries and contact times on larger surfaces emulating the disinfection process that occurs within a hospital setting. We hypothesized that fungicidal efficacies of disinfectant towelettes against *C. auris* will vary based on the formulation of the disinfectant used and surface area wiped. We also hypothesized that off-label contact time will significantly impact the overall disinfection efficacy of disinfectant towelettes against *C. auris*. Therefore, the objectives of our study were to (i) evaluate the efficacies of select disinfectant towelette products with varying chemistries on a hard, non-porous surface contaminated with *C. auris*, and (ii) determine if off-label contact times (e.g., 30 s, 1-, 2-, 3- and 10-min) influence the level of disinfection efficacy for all product types on a defined surface area. Our results demonstrated that overall hydrogen peroxide (HP)-based disinfectant towelette was significantly more fungicidal than the quaternary ammonium chloride (QAC)-alcohol-based disinfectant towelette. For all product types, 30 s contact time did not achieve the same level of disinfection as one-min or longer contact times. Overall, disinfectant towelette efficacy is dependent upon product formulation and contact time.

## Materials and methods

### Fungal strain, disinfectant towelette products, and surface type used in study

**Figure 1:**
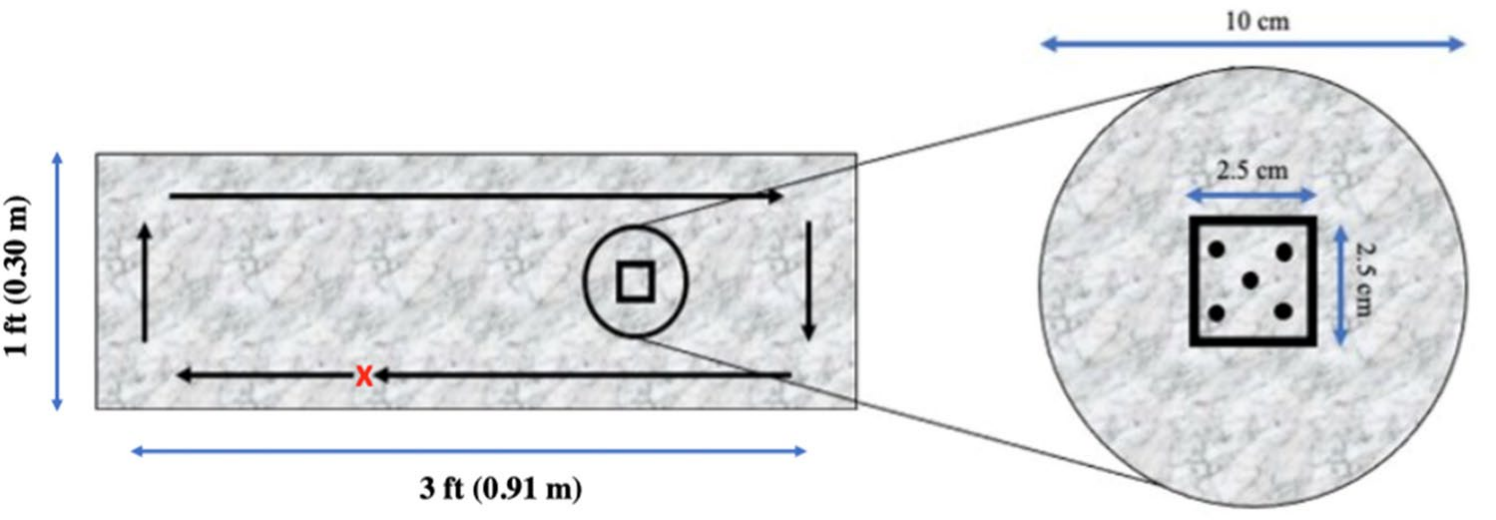
Schematic diagram of the Formica surface used for disinfectant towelette efficacy testing against *C. auris* Satoh et Makimura (ATCC MYA-5001). *Candida* *auris* cells were spot inoculated on the i-zone (black dots), marked within a square of 2.5 cm × 2.5 cm inscribed within a circular pattern of diameter 10 cm on the Formica sheet. After inoculation, the whole surface (3 ft^2^ or 0.28 m^2^) was wiped in a clockwise pattern as indicated by the black outlined arrows. Samples were collected from the marked circular zone post-disinfection to recover *C. auris*.

We tested fungicidal efficacies of five EPA registered disinfectant towelette products, with or without fungicidal claims (Table 1), against *C. auris* Satoh et Makimura (ATCC MYA-5001) contaminated test surface. The fungal test stock was prepared following a standard EPA protocol^17^. Irrespective of label contact time, all disinfectant towelette products were tested at 30 s, 1-min, 2-min, 3-min, and 10-min contact times against *C. auris* contaminated test surface following modifications to standard EPA methodology^18^. Of the total five disinfectant towelette products tested, three of the products were quaternary ammonium (QAC) i.e., QAC1, QAC2, and QAC3, one quaternary ammonium plus alcohol (QAC-alcohol), and one hydrogen peroxide (HP)-based disinfectant towelette product. QAC1, QAC2, QAC-alcohol, and HP were ready-to-use (RTU) towelette products while QAC3 was purchased as a concentrated solution and diluted at 1:256 with sterile hard water following EPA protocol^19^. Prior to use, 4.9 ml of QAC3 diluted solution was applied to a dry EasyWipe (Diversey Holdings Ltd., Fort Mill, SC). The test surface was imitation-granite surface laminated Formica approximately 3 ft (0.91 m) × 1 ft (0.09 m) to replicate countertop surfaces commonly found in healthcare facilities as shown in Fig. 1.

**Table 1:** Active ingredients, dilution at use, and label contact time of disinfectant products used in the study.

Disinfectant product^a^	Disinfectant Active Ingredient(s)^b^	Dilution at use	Label contact time (min)^c^
QAC^d^	0.14% alkyl dimethyl ethyl benzyl ammonium chloride + 0.14% alkyl dimethyl benzyl ammonium chloride	RTU	3
QAC2^d^	0.125% alkyl dimethyl ethyl benzyl ammonium chloride + 0.125% alkyl dimethyl benzyl ammonium chloride	RTU	3
QAC3^d^	6.510% octyl decyl dimethyl ammonium chloride + 2.604% dioctyl dimethyl ammonium chloride + 3.906% didecyl dimethyl ammonium chloride + 8.680% alkyl dimethyl benzyl ammonium chloride	1:256	10
QAC-alcohol^e^	0.25% alkyl dimethyl ethyl benzyl ammonium chloride + 0.25% alkyl dimethyl benzyl ammonium chloride + 55% isopropyl alcohol	RTU	2
HP^e^	0.5% hydrogen peroxide	RTU	1

^a^Abbreviated naming scheme for commercially available EPA registered disinfectant products used in this study;

^b^Active ingredients concentration;

^c^Defined label contact time for standard use;

^d^EPA registered disinfectant with no claims against *C. auris*;

^e^EPA registered disinfectant with claim against *C. auris.*

### Disinfection of the test surface, inoculation, and wiping method

The Formica surface was prepared using a five-step process to ensure sterility before and after *C. auris* disinfection for each trial. The surface was disinfected before cleaning followed by subsequent disinfection between replicates. Briefly, the test surface was treated with 10% bleach solution (Clorox, Oakland, CA) for 10 min, followed by rinsing with ultrapure water. Hydrogen peroxide (0.3% v/v; Equate, Freeport, TX) was applied for five min followed by rinsing with sterile ultrapure water. The test surface was cleaned using an all-purpose cleaner (Babyganics, Westbury, NY) followed by rinsing excess cleaner with ultrapure water and wiped dry. Lastly, the Formica sheet was wiped with 70% ethanol (Thermo Fisher, Waltham, MA) and allowed to evaporate completely before inoculating the surface within a 90 min period.

*Candida auris* inoculum was prepared following EPA MLB SOP-MB-35-0017 to test the efficacy of disinfectant towelettes at specific contact times. A soil load test suspension of 500 µL consisted of 25 µL of 5% bovine serum albumin (BSA; Fisher bioreagents, Ottawa, Canada), 35 µL of 5% yeast extract (ACROS Organics, New Jersey, US), 100 µL of 0.4% mucin stock (Abnova, Walnut, USA), and 340 µL of *C. auris* inoculum. Fungal suspension was then inoculated within the i-zone as depicted by black dots in the schematic diagram (Fig. 1) The i-zone comprised of a 2.5 × 2.5 cm square inscribed within a circle (10 cm in diameter) on the Formica sheet. Ten µl aliquots of *C. auris* test suspension were used to inoculate each black dot; totaling ~ 6 log_10_ CFU. Before disinfectant towelette testing, the first towelette was discarded from each RTU container and the second was used for wiping to ensure a consistent disinfectant liquid load on the towelettes. All wiping procedures were performed by the same individual to minimize variability. A single towelette (15.25 × 16.50 cm) was used to wipe the entire 0.28 m^2^ Formica sheet (Fig. 1) in a clockwise pattern with constant speed and pressure. Once the wiping procedure was complete, each disinfectant was left undisturbed for 30 s, 1-min, 2-min, 3-min, 10-min. This was followed by surface swabbing from the 10 cm diameter sampling zones using PUR-Blue swabs (World Bioproducts, Libertyville, IL) containing 10 ml sterile HiCap neutralizing buffer (World Bioproducts, Libertyville, IL) and stored at ambient room temperature before further processing or serial dilution, and subsequent plating. Three individual biological replications were conducted for each disinfectant towelette product at each contact time.

### Pathogen detection and enumeration

After sample collection, each PUR-Blue swab was vortexed for 30 s to release cells from the sponge into 10 mL sterile neutralizing buffer. The sponge swabs were removed from the vial, liquid aliquots were serially diluted followed by vacuum filtration onto sterile filter membranes (0.45 µm pore; Pall Corporation, Port Washington, NY). The filter membrane was aseptically transferred onto Sabourad Dextrose Agar (SDA) (VWR International, Radnor, PA) and incubated at 30 ± 2 °C for 48 ± 4 h.

### Statistical analyses

SAS v. 9.4 (SAS Institute, Cary, NC) was used to perform all statistical analyses. All CFU per cm^2^ from control and test samples were log_10_ transformed and normalized against control log_10_ densities to calculate log10 reductions. All fungicidal efficacy data were transformed into log_10_ reduction values for analyses to maximize fit; all analyses had a defined α = 0.05 significance level. To determine the factors significantly impacting fungal reduction, data were fitted to a linear mixed model with a post-hoc Dunnett’s t-test to analyze the significant differences between product performance at all contact times compared to one-min contact time. A separate least-square means comparison with Tukey adjustments was used to determine the significant differences among the five product types tested. Lastly, a separate Tukey studentized test was used to determine significant interactions between contact time and fungal log_10_ reduction (N = 75).

## Results

### Disinfectant towelette products vary in their fungicidal efficacies against *C*. *auris* and did not achieve standard six log_10_ reduction

**Figure 2:**
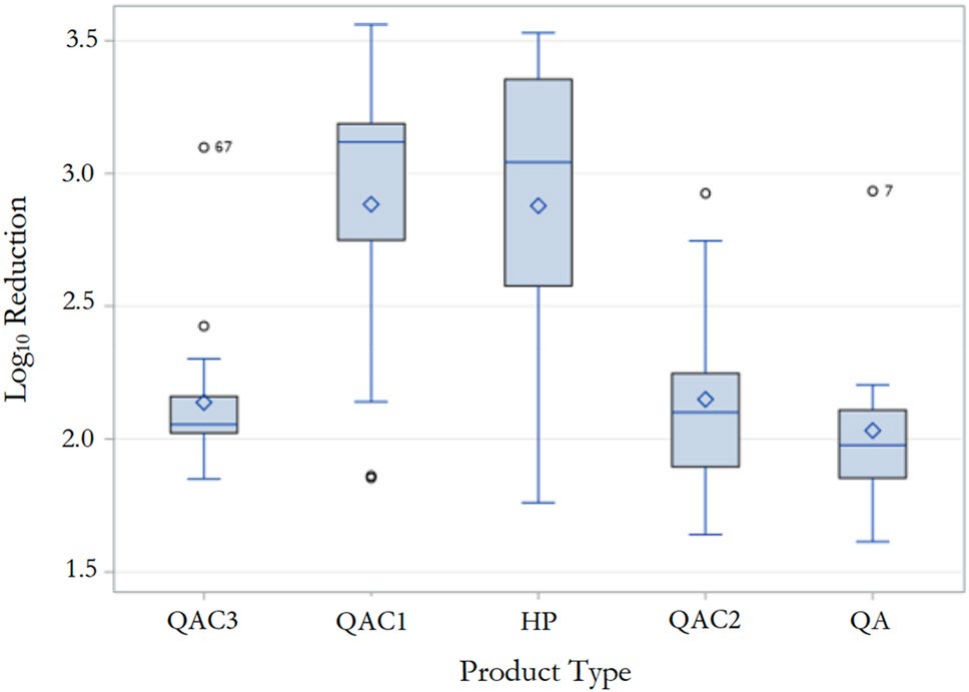
Mean log_10_ reductions for each product type against *C. auris* regardless of contact time. HP had the highest variability among products tested followed closely by QAC1. HP and QAC1 had significantly higher levels of fungicidal disinfect efficacies compared to QAC2, QAC3, and QAC-alcohol; (α = 0.05). ◇ Mean, — Median, ⊥ Q1-1.5 IQR, Τ Q3 + 1.5IQR, ○ Outlier.

Regardless of product type and chemistry, there were significant differences among the fungicidal efficacies of tested disinfectant towelette products against *C*. *auris* (*p* < 0.0001; Fig. 2). HP (2.82 ± 0.58) and QAC1 (2.81 ± 0.64) exhibited significantly higher fungal mean log_10_ reductions compared to other products irrespective of tested contact times (*p* < 0.0001). However, there was no significant difference in the fungicidal performances of QAC1- and HP-based disinfectant towelette product (*p* > 0.05). Among QAC products, QAC1 was significantly more fungicidal compared to QAC2 and QAC3 with mean fungal log_10_ reductions of 2.09 ± 0.32 and 2.03 ± 0.12, respectively (*p* < 0.05). There was no significant difference among the fungicidal efficacies of QAC2 (2.09 ± 0.32), QAC3 (2.03 ± 0.12), and QAC-alcohol (1.97 ± 0.17) disinfectant towelette products (*p* > 0.05; Fig. 2).

### At least one-min of contact time is required to achieve *C*. *auris* disinfection on a hard non-porous surface

**Figure 3:**
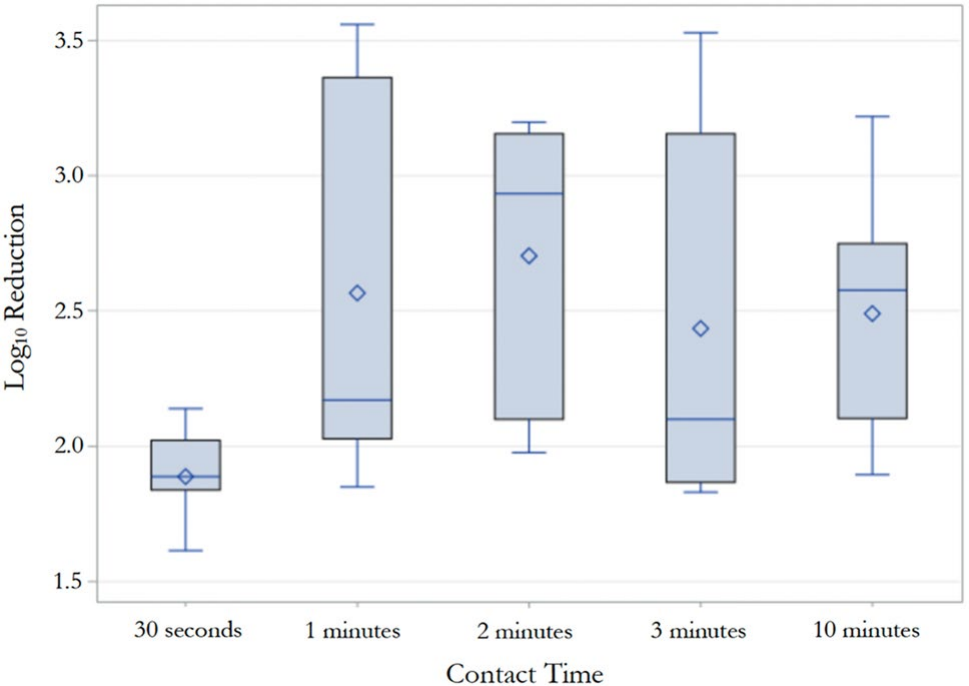
Effect of contact time on the disinfection efficacy against *C. auris* regardless of product type*.* 30 s of disinfection was significantly less effective than any of the other contact times i.e., one-min, two-min, three-min, and 10-min (p < 0.05). ◇ Mean, — Median, ⊥ Q1-1.5 IQR, Τ Q3 + 1.5IQR, ○ Outlier.

There were significant differences among tested contact times regardless of the product type (*p* < 0.0001; Fig. 3). Regardless of product type, 1-min contact time exhibited the highest mean log_10_ reduction (2.71 ± 0.66), while 30 s was least effective in reducing the fungal load on the Formica board with mean log10 reduction of 1.86 ± 0.15 (*p* < 0.05). There were no significant differences in disinfection efficacy between one-min (2.56 ± 0.66), 2-min (2.70 ± 0.51), 3-min (2.43 ± 0.64), and 10-min (2.49 ± 0.41) (*p* > 0.05) demonstrating that after one-min of disinfection is achieved, increasing the contact time does not significantly impact the disinfection efficacy under these test conditions (Fig. 3). However, irrespective of product type and product claim, contact times of 1-, 2-, 3-, and 10-min lead to significantly higher mean log_10_ reductions as compared to 30 s contact time (*p* < 0.05; Fig. 3).

**Figure 4:**
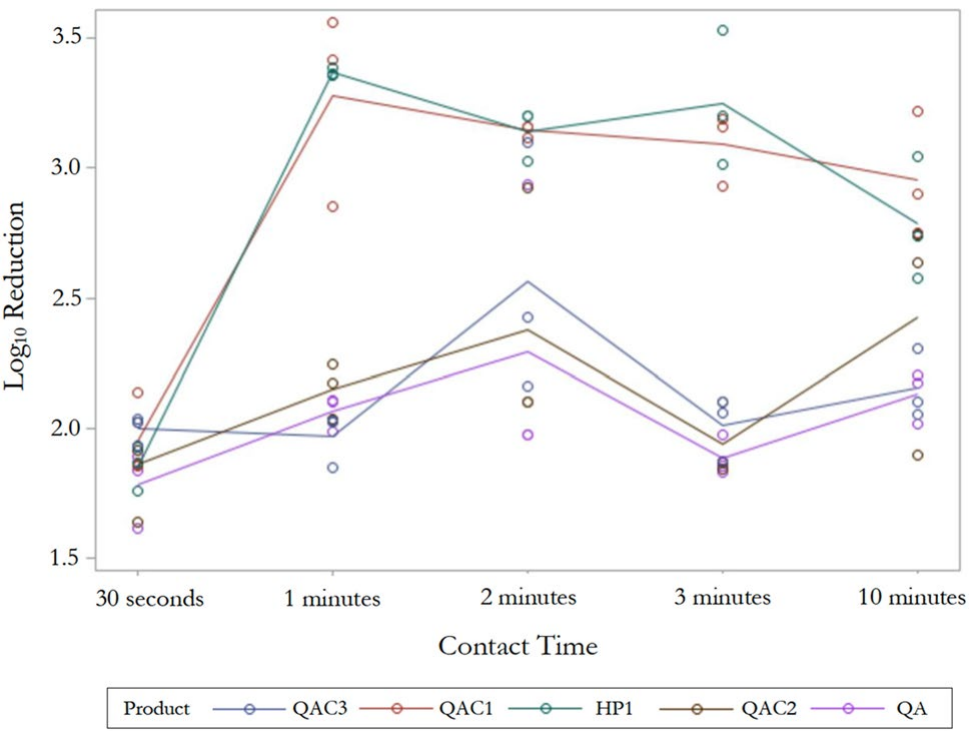
Interaction plot showing the relationship between contact time i.e., 30 s, one-min, two-min, three-min, and 10-min for each product (HP, QAC1, QAC2, QAC3, and QA) and mean log_10_ reductions against *C. auris.*

Both QAC1- and HP-based disinfectant towelette products exhibited the highest log_10_ reduction at most of the tested contact time points i.e., 30 s, 1-min, 2-min, and 3-min (Fig. 4). However, QAC1-based product efficacy was higher at 10-min as compared to HP-based disinfectant towelette product, but not statistically significant (*p* > 0.05). Performance of QAC2- and QA-based disinfectant towelette products at 30 s, one-min, two-min, and three-min were comparable (*p* > 0.05; Fig. 4), but there was an increase in the overall log_10_ reduction exhibited by QAC2 at 10-min contact time as compared to QA. For QAC3-based disinfectant towelette product, the maximum fungicidal efficacy was observed at two-min contact time although there were no significant differences among log10 reductions at all contact times tested (*p* > 0.05; Fig. 4). Overall, our findings further conclude that regardless of product chemistries, one-min of contact time is more effective than 30 s for adequate disinfection of *C. auris*.

## Discussion

QACs are among the most used disinfectant class in healthcare facilities for hard, non-porous surfaces due to their broad-spectrum antimicrobial activity and surfactant properties^20^. The discrepancy in QACs performance as observed in our study could be attributed to the varying concentrations of active ingredients used in the formulations. A study by Wesgate et al. demonstrated that QACs with side chains in the C12-16 range in their formulations were more adsorbed to different wipe material types than other formulations^16^. Considering that wipes are “wringed” to dispense disinfectant liquid, QACs may have been more adsorbed to the wipe material, resulting in an overall lower final disinfectant towelette-liquid concentration compared to HP product. In addition, the varying efficacy of QAC-based disinfectant towelettes against *C. auris* points towards the other factors impacting the efficacy and the scenarios in which disinfectant towelettes may not meet the required efficacy over larger defined surface areas.

Previous studies have shown that the addition of other active ingredients in quaternary ammonium-based products (e.g., quaternary ammonium plus alcohol-based products) increase the overall efficacy^21^; our findings suggest otherwise. A possible explanation could be that alcohol may be evaporating too quickly to achieve the higher log_10_ kill needed to meet the standard performance guidelines. We utilized larger surface areas to test the fungicidal efficacies of these disinfectant towelette products representing real-world disinfection scenarios, which is an important test of product performance. These trends are consistent with our previous findings testing disinfectant towelette product efficacies against bacterial cultures of *P. aeruginosa* and *S. aureus* contaminated Formica sheet^10^. No peer reviewed literature is currently available on disinfectant towelette efficacies against *C. auris* on larger surfaces such as Formica used in our study.

Hydrogen peroxide is a strong oxidizing agent and is known to be highly efficacious against bacteria^10,22^ and fungi^23^. HP-based product achieved the highest log_10_ reductions; however, there was a lot of variability in off-and on-label contact time evaluation. Although our findings on higher efficacy values for the HP-based product against *C. auris* are consistent with previous findings by Sexton et al.^23^ and Cadnum et al.^24^, we did not achieve the six log_10_ reduction standard performance. These studies followed the American Society for Testing and Materials (ASTM) Standard quantitative carrier disk test method (ASTM E2197-11)^25^ which use smaller surfaces i.e., 1 cm^2^ stainless steel carrier disks to test the efficacies of disinfectants. It is important to evaluate disinfectants performances based on how they will be used in the field. There is currently no guidance on the total area that can be effectively disinfected with a single towelette. Liquid load, concentration of the active ingredient(s), mechanical force, and towelette material collectively impact disinfectant efficacy. In our previous study, we demonstrated that disinfectant towelettes post-disinfection retained viable cells and could become a potential reservoir for recontamination if the antimicrobial agents do not fully inactivate the viable pathogens within the towelette substrate^26^. Our results corroborate a growing body of knowledge on inefficacy of disinfectant towelette regardless of label contact time, type of active ingredient, and registered six-log_10_ reduction claim against inactivating *C. auris* from larger surfaces.

Findings by Sexton et al. showed that label contact time of 10 min was only able to achieve a 0.56 log_10_ reduction^23^. This low level of fungicidal efficacy suggests that mechanical removal also plays a significant role in the primary mechanism responsible for *C. auris* reduction over larger surfaces. In our previous work, we showed how unimpregnated towelettes with the addition of phosphate buffered saline (PBS) were able to achieve a ~ 3 log_10_ reduction of bacterial CFU over a larger surface area^10^. Similar results can be seen here with disinfectant solutions that were not as effective with a less than three log_10_ reduction of *C. auris*^23^. Mechanical removal and shear stress from wiping are also independent of contact time, which would enable reduced efficacy between two and three min of contact time and contribute to variation. Most studies to-date are evaluating the efficacies of different disinfectant product types against *C. auris* on smaller surfaces, regardless of contact time. Our study is the first to demonstrate the effect of different label contact times on the efficacies of disinfectant products which in turn, represent variations in contact time and chemistry used in the healthcare environment.

## Conclusions

Overall, the fungicidal efficacies of disinfectant towelettes, with or without claims against *C. auris*, vary among product types and label contact times. HP-based disinfectant towelettes were significantly more effective as compared to QAC-alcohol-based disinfectant towelettes. Irrespective of the product type used, 30 s of contact time was significantly less effective in reducing *C. auris* from the surface compared to one-min and longer contact times. However, there was no significant additional reduction in *C. auris* after prolonged contact times i.e., 2-, 3-, 10-min as compared to one-min contact time, regardless of disinfectant type. None of the tested disinfectant towelettes used in this study were able to achieve a standard six-log_10_ reduction of *C. auris* over the tested surface area. In conclusion, our findings indicate that while selection of active ingredients class for disinfectants is crucial for effective disinfection, larger surface area and label contact time also impact efficacy.

## Supplementary Information

Supplementary Table 1.

## Data Availability

All data generated or analyzed during this study are included in this published article [and its supplementary information files].
