# Supplementary material for: Contact time and disinfectant formulation significantly impact the efficacies of disinfectant towelettes against Candida auris on hard, non-porous surfaces
Source: Sci Rep. 2023 Apr 10;13:5849. doi: 10.1038/s41598-023-32876-y (PMC10086017; doi:10.1038/s41598-023-32876-y)
Supplement: Supplementary file 1 — Supplementary Table 1. [file 41598_2023_32876_MOESM1_ESM.docx]

**Table 1.** Evaluation of disinfectant efficacy for product type

and contact time against *C. auris* over a large surface area

| **Product^a^** | **Time^b^** | **LogReduction^c^** |
| --- | --- | --- |
| QAC-alcohol | 30 second | 1.888193017 |
| QAC-alcohol | 30 second | 1.838290898 |
| QAC-alcohol | 30 second | 1.614327273 |
| QAC2 | 30 second | 2.022711348 |
| QAC2 | 30 second | 1.91688327 |
| QAC2 | 30 second | 1.640234697 |
| QAC | 30 second | 1.861902964 |
| QAC | 30 second | 2.139910568 |
| QAC | 30 second | 1.853700368 |
| HP | 30 second | 1.865652292 |
| HP | 30 second | 1.926161895 |
| HP | 30 second | 1.761069153 |
| QAC3 | 30 second | 2.023483213 |
| QAC3 | 30 second | 2.035343548 |
| QAC3 | 30 second | 1.932942564 |
| QAC-alcohol | 1 minute | 2.098464581 |
| QAC-alcohol | 1 minute | 1.987246674 |
| QAC-alcohol | 1 minute | 2.109161292 |
| QAC2 | 1 minute | 2.247453447 |
| QAC2 | 1 minute | 2.171405555 |
| QAC2 | 1 minute | 2.034585545 |
| QAC | 1 minute | 3.417406166 |
| QAC | 1 minute | 2.850480731 |
| QAC | 1 minute | 3.559630352 |
| HP | 1 minute | 3.353737086 |
| HP | 1 minute | 3.386520922 |
| HP | 1 minute | 3.363335707 |
| QAC3 | 1 minute | 2.02407773 |
| QAC3 | 1 minute | 2.028394688 |
| QAC3 | 1 minute | 1.849898529 |
| QAC-alcohol | 2 minute | 2.933391635 |
| QAC-alcohol | 2 minute | 1.97735134 |
| QAC-alcohol | 2 minute | 1.97735134 |
| QAC2 | 2 minute | 2.924534201 |
| QAC2 | 2 minute | 2.10049176 |
| QAC2 | 2 minute | 2.10049176 |
| QAC | 2 minute | 3.117916062 |
| QAC | 2 minute | 3.1552484 |
| QAC | 2 minute | 3.1552484 |
| HP | 2 minute | 3.026884913 |
| HP | 2 minute | 3.19800038 |
| HP | 2 minute | 3.19800038 |
| QAC3 | 2 minute | 3.097422469 |
| QAC3 | 2 minute | 2.160711299 |
| QAC3 | 2 minute | 2.424869935 |
| QAC-alcohol | 3 minute | 1.97735134 |
| QAC-alcohol | 3 minute | 1.853923338 |
| QAC-alcohol | 3 minute | 1.830663704 |
| QAC2 | 3 minute | 2.100491753 |
| QAC2 | 3 minute | 1.867120075 |
| QAC2 | 3 minute | 1.843339292 |
| QAC | 3 minute | 3.1552484 |
| QAC | 3 minute | 2.931578064 |
| QAC | 3 minute | 3.187277837 |
| HP | 3 minute | 3.198000381 |
| HP | 3 minute | 3.529090427 |
| HP | 3 minute | 3.011186578 |
| QAC3 | 3 minute | 2.103497011 |
| QAC3 | 3 minute | 2.057497069 |
| QAC3 | 3 minute | 1.87095524 |
| QAC-alcohol | 10 minute | 2.203707975 |
| QAC-alcohol | 10 minute | 2.171043891 |
| QAC-alcohol | 10 minute | 2.017913925 |
| QAC2 | 10 minute | 1.895396225 |
| QAC2 | 10 minute | 2.632265735 |
| QAC2 | 10 minute | 2.745916802 |
| QAC | 10 minute | 3.218648325 |
| QAC | 10 minute | 2.748771304 |
| QAC | 10 minute | 2.898526965 |
| HP | 10 minute | 2.734348486 |
| HP | 10 minute | 2.576407918 |
| HP | 10 minute | 3.041617964 |
| QAC3 | 10 minute | 2.103497011 |
| QAC3 | 10 minute | 2.055046883 |
| QAC3 | 10 minute | 2.302527849 |

^a^ Disinfectant wipes active ingredient;

^b^ The amount of fungal load on the board after the wiping procedure has been performed compared to an unwiped contaminated surface;

^c^ Contact time the liquid load was left undisturbed on the surface before swabbing.
